# Hyponatremia as the Initial Presentation of Rathke’s Cleft Cyst-Related Hypopituitarism

**DOI:** 10.7759/cureus.114171

**Published:** 2026-08-08

**Authors:** Naung Latt Htun, Aye Nyein Thinzar, Hsan Theingi Win, Dilip Joseph Thottacherry

**Affiliations:** 1 Internal Medicine, Suri Seri Begawan Hospital, Kuala Belait, BRN; 2 Radiology, Raja Isteri Pengiran Anak Saleha Hospital, Bandar Seri Begawan, BRN

**Keywords:** anterior pituitary hormone, hypopituitarism, hypotonic hyponatremia, low cortisol, rathke’s cleft cyst

## Abstract

Rathke’s cleft cyst (RCC) is a benign sellar lesion that is frequently detected incidentally, while symptomatic cases are uncommon. Endocrine dysfunction may occur due to compression of the pituitary gland, but profound hyponatremia as the initial manifestation of RCC-related hypopituitarism is rare and may delay diagnosis. We report the case of a 76-year-old woman who presented with persistent dizziness and severe euvolemic hyponatremia. Further endocrine assessment demonstrated secondary adrenal insufficiency, central hypothyroidism, hypogonadotropic hypogonadism, and mild hyperprolactinemia. Magnetic resonance imaging revealed an RCC with mild suprasellar extension and pituitary stalk deviation. The patient was treated with hydrocortisone followed by levothyroxine, resulting in clinical improvement and normalization of sodium levels. Although transsphenoidal surgery was recommended, she declined operative management and remained clinically stable with hormone replacement therapy during six months of follow-up. This case highlights that RCC-related hypopituitarism should be considered in patients presenting with unexplained euvolemic hyponatremia, even in the absence of headache, visual disturbances, or other classical sellar symptoms. Early endocrine evaluation and pituitary imaging are essential to establish the diagnosis, initiate appropriate hormone replacement, and prevent recurrent hyponatremia and potentially life-threatening adrenal insufficiency.

## Introduction

Rathke’s cleft cyst (RCC) is a benign, non-neoplastic epithelial cyst arising from persistent remnants of Rathke’s pouch, an embryologic structure that gives rise to the anterior pituitary gland [1,2]. Autopsy studies suggest that RCCs are relatively common, with a prevalence ranging from 13% to 22% [3]; however, most lesions remain asymptomatic throughout life and are detected incidentally during neuroimaging performed for unrelated indications [4]. Symptomatic RCCs are uncommon and typically occur when cyst enlargement compresses adjacent structures, resulting in headaches, visual disturbances, endocrine dysfunction, or, less frequently, pituitary apoplexy-like presentations [1,5]. Although RCCs are generally considered benign lesions, their location within the sellar region can lead to significant morbidity through compression of the pituitary gland and stalk, causing varying degrees of hypopituitarism and hyperprolactinemia [2,4].

Endocrine dysfunction is observed in a subset of patients with symptomatic RCCs and may involve isolated or multiple anterior pituitary hormone deficiencies [4,6]. Secondary adrenal insufficiency is among the most clinically significant manifestations because delayed recognition can result in life-threatening complications [7,8]. Cortisol deficiency impairs renal free-water excretion by removing the inhibitory effect of cortisol on arginine vasopressin secretion, leading to water retention and dilutional hyponatremia [9]. Consequently, severe hyponatremia may be the first clinical manifestation of previously unrecognized hypopituitarism.

Although hyponatremia has been reported in patients with pituitary disorders, it is an uncommon initial presentation of RCC-related hypopituitarism, particularly in the absence of headache, visual impairment, or other classical symptoms of sellar masses [6]. Early recognition is crucial because prompt glucocorticoid replacement rapidly corrects both the underlying endocrine deficiency and the associated electrolyte disturbance, while failure to identify secondary adrenal insufficiency may lead to recurrent hyponatremia and adrenal crisis [10].

We report the case of a 76-year-old woman who presented with profound symptomatic hyponatremia as the initial manifestation of panhypopituitarism secondary to an RCC. This case highlights the importance of considering central adrenal insufficiency and hypopituitarism in the differential diagnosis of unexplained euvolemic hyponatremia and underscores the value of comprehensive endocrine evaluation in identifying occult sellar pathology.

## Case presentation

The patient was a 76-year-old woman with a history of hypertension and hypercholesterolemia, taking amlodipine 5 mg once daily and atorvastatin 20 mg at bedtime. She presented to the emergency department with two days of dizziness without associated nausea, vomiting, or fainting. Examination showed a blood pressure of 141/53 mmHg with no postural drop, a heart rate of 63 beats/minute, a normal blood glucose level of 5.6 mmol/L, normal heart sounds, and vesicular breath sounds. The central nervous system examination was unremarkable, with no cerebellar abnormalities. She was discharged with a diagnosis of peripheral vertigo after symptomatic treatment. The following week, she continued to experience dizziness and was admitted to the medical ward. Initial tests revealed hyponatremia, with sodium at 112 mmol/L. Serum osmolality was 241 mOsm/kg, urine osmolality was 244 mOsm/kg, and urine sodium was 63 mmol/L. An 8 am cortisol level was 32 nmol/L. The standard high-dose (250 µg) short synacthen test confirmed hypocortisolism, with cortisol levels of 93 nmol/L at 0 minutes, 301 nmol/L at 30 minutes, and 365 nmol/L at 60 minutes after synacthen. Further evaluation showed low adrenocorticotropic hormone (ACTH) at 3.4 pg/mL, low-normal thyroid-stimulating hormone (TSH) at 0.661 uIU/mL, low free T4 at 7.2 pmol/L, low follicle-stimulating hormone and luteinizing hormone levels, low-normal insulin-like growth factor 1, and a mildly elevated prolactin level (Table 1).

**Table 1:** Laboratory investigations. ACTH: adrenocorticotropic hormone; TSH: thyroid-stimulating hormone; T3: triiodothyronine; T4: thyroxine; FSH: follicle-stimulating hormone; LH: luteinizing hormone; IGF-1: insulin-like growth factor 1

Tests	Values	Units	Reference range
Sodium	112	mmol/L	136–145
Potassium	3.3	mmol/L	3.5–5.1
Bicarbonate	23	mmol/L	22–29
Urea	0.4	mmol/L	2.8–8.1
Creatinine	49	µmol/L	50.4–98.1
Calcium	2.42	mmol/L	2.15–2.5
Phosphate	1.36	mmol/L	0.81–1.45
Uric acid	282	µmol/L	142.8–339.2
Blood glucose	5.6	mmol/L	4.0–7.8
Serum osmolality	241	mOsm/kg	275–305
Urine osmolality	244	mOsm/kg	50–1,200
Urine sodium	63	mmol/L	40–220
8 am cortisol	32	nmol/L	102.1–535.2
Synacthen test		nmol/L	-
0 minute	93		
30 minutes	301		
60 minutes	365		
ACTH	3.4	pg/mL	7.2–63.3
Prolactin	36.8	ng/mL	5.2–26.5
TSH	0.661	µIU/mL	0.27–4.2
Free T3	4.41	pmol/L	3.1–6.8
Free T4	7.2	pmol/L	12–22
FSH	1.7	mIU/mL	26.72–133.41
LH	<0.1	mIU/mL	5.16–61.99
IGF-1	59.8	ng/mL	53.5–160

**Figure 1:**
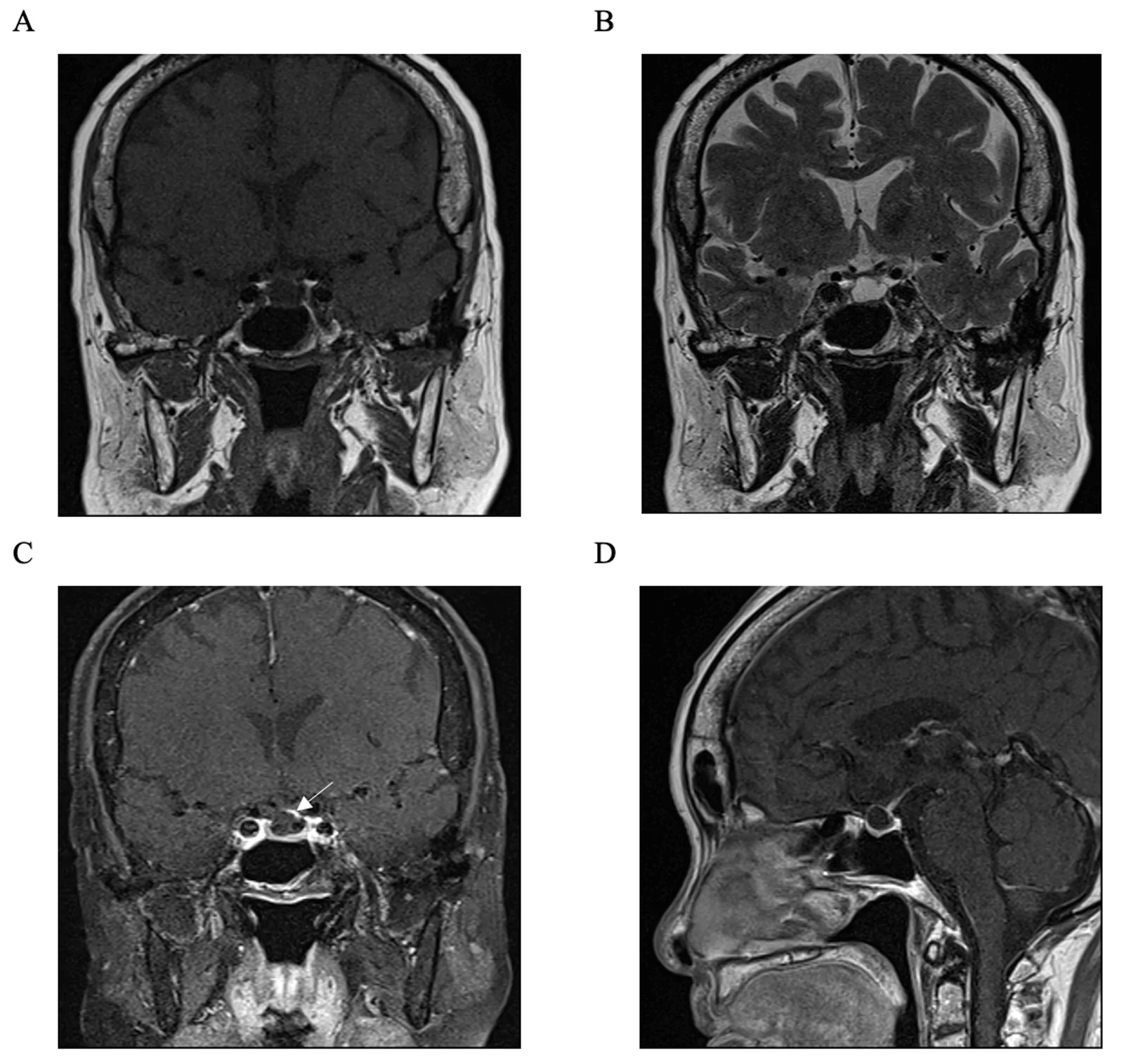
Magnetic resonance imaging (MRI) sequences, including coronal T1-weighted (A), coronal T2-weighted (B), and post-contrast images in coronal and sagittal (C, D) demonstrate a Rathke’s cleft cyst causing mild compression and leftward displacement of the pituitary stalk (arrow).

Magnetic resonance imaging (MRI) of the brain was performed to investigate secondary causes of hypoadrenalism, revealing a lesion with hypointense signal on T1-weighted imaging and hyperintense signal on T2-weighted imaging, without a discrete enhancing wall or intracystic nodule after contrast administration. These findings were consistent with an RCC (0.7 cm × 1 cm × 0.8 cm) in the pituitary fossa with slight suprasellar extension without optic chiasmal compression and a leftward deviation of the pituitary stalk (Figure 1).

Eye examination showed no visual field defects. She was diagnosed with hypopituitarism due to an RCC. She was started on hydrocortisone 10 mg twice daily, followed by thyroxine 75 µg once daily, and referred to neurosurgery for transsphenoidal pituitary surgery. Unfortunately, she refused the surgery and continued on hormone replacement for hypopituitarism. She did not experience any compressive symptoms and had stable hormone tests and imaging findings at a six-month follow-up.

## Discussion

RCC is a benign epithelial cyst derived from embryological remnants of Rathke’s pouch and represents one of the most common cystic lesions of the sellar region [1]. Although RCCs are identified in up to one-third of autopsy specimens, the vast majority remain clinically silent throughout life [2]. Symptomatic RCCs are relatively uncommon and typically result from progressive cyst enlargement that compresses the pituitary gland, pituitary stalk, or optic apparatus. The most frequent clinical manifestations include headache, visual impairment, endocrine dysfunction, and, less commonly, pituitary apoplexy-like presentations [1,5]. Endocrine abnormalities result from compression of normal pituitary tissue or interruption of hypothalamic-pituitary signaling, leading to deficiencies in one or more anterior pituitary hormones [2]. Despite these recognized manifestations, profound hyponatremia as the initial presentation of RCC-associated hypopituitarism remains uncommon and may delay diagnosis if the underlying endocrine disorder is not considered.

Our patient initially presented with isolated dizziness and was discharged with a presumed diagnosis of peripheral vertigo because she lacked classic features of adrenal insufficiency or a sellar mass, such as headache, visual disturbance, hypotension, or altered consciousness. Only after persistent symptoms and the discovery of profound hyponatremia was further endocrine evaluation undertaken, which ultimately revealed secondary adrenal insufficiency, central hypothyroidism, hypogonadotropic hypogonadism, and MRI evidence of an RCC. This case illustrates the nonspecific nature of hypopituitarism in older adults and shows how endocrine disease may initially masquerade as more common neurological or vestibular disorders.

Hyponatremia is one of the most important biochemical manifestations of secondary adrenal insufficiency and may be the presenting feature of hypopituitarism [9]. The mechanism differs from that observed in primary adrenal insufficiency. In secondary adrenal insufficiency, aldosterone secretion is largely preserved because it is primarily regulated by the renin-angiotensin-aldosterone system [10]. Instead, cortisol deficiency impairs suppression of corticotropin-releasing hormone and arginine vasopressin (antidiuretic hormone), leading to persistent vasopressin secretion despite hypo-osmolality [8]. Increased vasopressin promotes renal free-water retention, causing dilutional hyponatremia. Furthermore, cortisol deficiency reduces glomerular filtration rate and impairs renal free-water clearance, thereby exacerbating water retention [8]. Concomitant central hypothyroidism may also contribute to impaired water excretion [11], although adrenal insufficiency remains the predominant mechanism in most patients with hypopituitarism. Consequently, the biochemical profile frequently resembles the syndrome of inappropriate antidiuretic hormone secretion (SIADH), characterized by hypotonic hyponatremia with inappropriately concentrated urine and elevated urine sodium [12]. Recognition of this physiological distinction is essential because glucocorticoid replacement, rather than fluid restriction alone, is the definitive treatment.

Our patient’s biochemical findings were highly consistent with this mechanism. She had severe hypotonic hyponatremia with inappropriately elevated urine osmolality and urine sodium, findings that could easily have been misinterpreted as SIADH. However, the markedly reduced morning cortisol concentration, low ACTH level, and inadequate response to the short Synacthen test confirmed secondary adrenal insufficiency. Additional hormonal evaluation demonstrated central hypothyroidism, hypogonadotropic hypogonadism, and low-normal insulin-like growth factor-1, indicating multiple anterior pituitary hormone deficiencies. Mild hyperprolactinemia was likely attributable to pituitary stalk compression, resulting in reduced dopaminergic inhibition of prolactin secretion [9]. These endocrine findings, together with the MRI demonstration of a sellar cyst causing pituitary stalk deviation, strongly supported RCC-related hypopituitarism as the underlying cause.

Previous studies have demonstrated that endocrine dysfunction is not uncommon in patients with symptomatic RCC. In a retrospective cohort of 221 patients, Zhang et al. reported pituitary dysfunction in approximately one-quarter of patients, including central hypothyroidism, hypogonadotropic hypogonadism, growth hormone deficiency, and secondary adrenal insufficiency [3]. Interestingly, pituitary dysfunction was not consistently associated with cyst size, suggesting that the anatomical relationship between the cyst and the normal pituitary gland may be more important than lesion diameter alone [3]. Therefore, comprehensive endocrine evaluation should be performed in all patients with RCC regardless of lesion size. Our patient’s cyst measured only 0.7 × 1.0 × 0.8 cm yet produced deficiencies involving multiple pituitary axes, illustrating that relatively small lesions can cause clinically significant hypopituitarism.

Several published case reports describe RCC presenting with panhypopituitarism and hyponatremia; however, most patients had more obvious symptoms such as headache, visual impairment, nausea, vomiting, or altered mental status. Mukherjee et al. reported two patients whose persistent hyponatremia ultimately led to the diagnosis of panhypopituitarism secondary to RCC after extensive investigation for gastrointestinal symptoms [6]. Our patient differed in that dizziness secondary to profound hyponatremia was the only presenting complaint, without headache, visual field defects, or symptoms suggesting an expanding sellar mass. This atypical presentation emphasizes the importance of maintaining a high index of suspicion for endocrine disorders in patients with otherwise unexplained euvolemic hyponatremia.

The management of symptomatic RCC remains controversial and should be individualized according to the patient’s clinical presentation. Surgical decompression via transsphenoidal surgery is generally recommended for patients with progressive visual impairment, optic chiasmal compression, intractable headache, or significant neurological deficits [13]. Surgery frequently improves headaches and visual symptoms, although recovery of pituitary function is less predictable because prolonged compression may result in irreversible damage to the anterior pituitary [4,14]. Conversely, patients without visual compromise or neurological deterioration may be managed conservatively with hormone replacement therapy and serial clinical and radiological follow-up [13]. Recent reports have demonstrated favorable outcomes with conservative management in carefully selected patients, particularly when hormonal deficiencies are adequately replaced, and the cyst remains radiologically stable [15].

Our patient had no visual field defects despite slight suprasellar extension of the cyst and was therefore referred for elective neurosurgical evaluation rather than emergency intervention. Although transsphenoidal surgery was recommended, she declined operative treatment because of her age and personal preference. She was therefore managed conservatively with physiological hydrocortisone replacement followed by levothyroxine after glucocorticoid therapy had been established. This treatment sequence is consistent with current Endocrine Society recommendations, which emphasize correcting adrenal insufficiency before initiating thyroid hormone replacement to avoid precipitating adrenal crisis by increasing cortisol metabolism [7]. Long-term endocrine follow-up and periodic MRI surveillance remain essential because pituitary hormone deficiencies often persist despite conservative treatment and cyst enlargement may occur during follow-up.

This case highlights several important clinical lessons. First, severe euvolemic hyponatremia should always prompt evaluation for adrenal insufficiency before a diagnosis of SIADH is established. Second, a normal or low-normal TSH does not exclude central hypothyroidism, particularly in the presence of low free thyroxine concentrations and other pituitary hormone deficiencies. Third, RCC should be included in the differential diagnosis of unexplained hypopituitarism even when the lesion is relatively small and classical compressive symptoms are absent. Finally, comprehensive endocrine assessment in patients with sellar lesions is essential because hormonal abnormalities may be the only indication of clinically significant pituitary dysfunction. Early recognition and appropriate hormone replacement can rapidly reverse life-threatening endocrine complications while preventing recurrent hyponatremia and adrenal crisis.

## Conclusions

RCC should be considered a potential cause of hypopituitarism in patients presenting with unexplained euvolemic hyponatremia, even in the absence of headache or visual symptoms. Early endocrine evaluation and pituitary imaging are essential for timely diagnosis, while prompt hormone replacement therapy can rapidly correct electrolyte abnormalities and prevent life-threatening complications.
